# Workload Pressure Among Nurses in Central Europe: Implications for Human Resource Management in Poland, the Czech Republic, and Slovakia

**DOI:** 10.1155/jonm/9303525

**Published:** 2026-05-27

**Authors:** Katarzyna Tomaszewska, Bożena Majchrowicz, Helena Kadučáková, Anna Krátká, Erika Durejova, Krzysztof Kalita, Krystyna Kowalczuk

**Affiliations:** ^1^ Department of Nursing, Institute of Health Protection, The Bronisław Markiewicz Academy of Applied Sciences, Jarosław, Poland; ^2^ Department of Health Sciences and Psychology, Institute of Nursing, University of Rzeszów, Rzeszów, Poland, ur.edu.pl; ^3^ Department of Nursing, Faculty of Health Sciences, Catholic University in Ružomberok, Ružomberok, 034 01, Slovakia, ku.sk; ^4^ Department of Health Care Sciences, Faculty of Humanities, Tomas Bata University in Zlin, Zlín, 760 01, Czech Republic, utb.cz; ^5^ Ustredna Vojenska Nemocnica, Ružomberok, Slovakia; ^6^ Bureau of Statistical Research and Analysis, Rzeszow, Poland; ^7^ Department of Integrated Medical Care, Medical University of Białystok, Białystok, Poland, umb.edu.pl

**Keywords:** KONOP questionnaire, nurse, occupational stress, work overload, workload

## Abstract

**Introduction:**

Nurses constitute the largest professional group in the healthcare system. Their work, which requires constant contact with patients, families, and medical staff, involves the risk of excessive psychological and physical strain. Understanding the mechanisms of work overload is crucial both for patient safety and for the professional well‐being of staff.

**Aim:**

The aim of this study was to examine whether excessive workload is present among professionally active nurses in Poland, Slovakia, and the Czech Republic.

**Material and methods:**

The study was conducted among three groups of nurses employed in hospitals in Poland, Slovakia, and the Czech Republic. The survey was carried out from September 2024 to the end of March 2025. The study sample included 260 Polish nurses, 236 Slovak nurses, and 258 Czech nurses. The research tool consisted of a proprietary questionnaire covering sociodemographic data, as well as the standardized KONOP Work Overload Symptom Questionnaire. The chi‐square test for independence was used for statistical analysis. To assess the strength of associations, Phi and Cramér’s *V* coefficients were applied. The Mann–Whitney *U* test was used for nonparametric comparison of differences. Correlations between ordinal or quantitative variables were analyzed using Spearman’s rho coefficient.

**Results:**

Nurses from Poland obtained the highest scores for loss of control over work, perfectionistic work style, and perceived organizational oppressiveness. The highest scores for general attitudes toward work were recorded among Slovak nurses. Statistically significant differences between the groups were found across all scales of the KONOP questionnaire.

**Implications for Nursing Management:**

The country of professional practice differentiates the level of excessive workload among nurses. The greater intensity of selected dimensions of workaholism among Polish nurses may indicate a higher organizational burden and stronger occupational pressure. The findings suggest the need to implement preventive measures aimed at reducing work overload and improving the organizational conditions of nurses’ work environments.

## 1. Introduction

Nursing is one of the most demanding professions in the healthcare system. Due to the complex nature of the duties performed, the need for multitasking, and constant exposure to human suffering, nurses are particularly vulnerable to stress and work overload. According to their professional competencies, nurses are responsible for performing medical procedures, providing patient care, maintaining documentation, operating medical equipment, conducting physical examinations, and collaborating with the therapeutic team [1].

A significant source of stress among nursing staff is the unique organization of their work, characterized by irregular hours, shift work, prolonged periods of tension, and staff shortages. These factors collectively lead to physical and mental exhaustion. Time pressure, in turn, contributes to progressive fatigue, reduced work efficiency, and a decline in nurses’ psychological well‐being [2].

According to the World Health Organization (WHO), workers’ health includes not only physical safety but also psychological well‐being and job satisfaction. Psychosocial factors such as irregular working hours, staffing shortages, and the pressure of responsibilities lead to exhaustion, reduced effectiveness, and job dissatisfaction. Psychosocial occupational hazards were defined as “those aspects of work organization and management, along with their social and environmental context, that may potentially cause psychological, social, or physical harm” [3, 4].

Workload is one of several variables that can impact the quality of nursing care. Excessive workload can compromise both the safety of patients and the quality of nursing services in hospital settings [5, 6]. Nursing workload is defined as the level of essential clinical skills required to perform routine nursing duties. These required skills vary depending on the type of clinical care and the hospital department where nurses are assigned [7].

The way individuals subjectively perceive their workload—regardless of the actual volume—is referred to as workload perception. When a person believes their available resources are sufficient to meet job demands, the workload is perceived as manageable. If they believe their resources are insufficient, the workload is seen as high. Conversely, when resources exceed the demands, the workload may be perceived as low [8].

Research has identified a correlation between high nursing workload and increased patient mortality in hospitals. In addition to higher mortality rates, these studies have also found a direct link between increased patient‐to‐nurse ratios and higher levels of nurse burnout [9, 10]. Excessive workload has also been associated with a higher incidence of patient falls, medication errors, elevated infection risks, and a general decline in patient safety. Conversely, improvements in nurse‐to‐patient ratios and reduced nursing workload have been linked to better patient outcomes [11].

Despite numerous studies on measuring nursing workload, it remains a prominent topic in the nursing literature. It has been described as both “the total volume of nursing work that must be completed within a specific time frame” and “the amount of time and care a nurse dedicates to direct or indirect patient care, workplace responsibilities, and professional development.” Systems designed to quantify patient care needs—considering patient acuity, care complexity, case mix, and patient turnover—aim to estimate the required nursing resources and the associated workload [12].

An increase in the number of patients per nurse, along with heightened patient demands and complex diagnoses, can create a mismatch between patient needs and available nursing resources, resulting in a greater workload [13]. Additionally, higher patient volumes and demands limit nurse–patient interaction, lead to unfinished tasks, heighten time pressure, and raise concerns about patient outcomes [14].

Workload includes both physical and mental elements that interact and influence an individual’s performance. Physical workload relates to manual tasks, such as lifting patients and administering medications. Mental workload involves receiving, processing, and interpreting information, making decisions, maintaining focus, and engaging with patients and their families. Literature suggests that these physical and mental components often influence each other. Mental workload, which involves subjective processes, can affect physical abilities by causing fatigue and functional errors. Conversely, increased physical effort demands additional cognitive resources, potentially diminishing concentration and precision and lowering nurse productivity over time [15].

In the scientific literature, nurses’ workload is often analyzed within the framework of psychosocial work models, particularly the job demands–resources (JD‐R) model. This model assumes that high job demands—such as time pressure, a large number of patients, and emotional burden—combined with insufficient organizational resources (e.g., team support, autonomy, or adequate staffing levels), lead to work overload, emotional exhaustion, and professional burnout [16]. In the nursing environment, these factors may affect not only staff well‐being but also patient safety and the quality of healthcare [17].

Mental workload is a complex, multifaceted construct influenced by external job demands, the work environment, psychological and organizational factors, and cognitive and structural capabilities. As a critical element of healthcare service delivery, workload plays a pivotal role in negative outcomes such as emotional exhaustion, depersonalization, and burnout. Excessive occupational stress can pose a threat to workers’ health, resulting in physical, mental, and behavioral issues. Employees in poor mental health are less capable of delivering high‐quality care. Thus, ensuring the mental well‐being of healthcare staff is essential for delivering safe and effective patient care. Poor mental health among healthcare professionals can further lead to professional challenges and diminished performance [18].

Healthcare systems in Central European countries such as Poland, the Czech Republic, and Slovakia differ in terms of the organization of nursing work, employment structures, and methods of determining nurse staffing levels [19]. Nursing education in these countries is currently aligned with European Union standards and is primarily provided at the higher education level within the Bologna system, in accordance with the requirements of Directive 2005/36/EC.

In Poland, legally defined minimum staffing standards for nurses and midwives are in place, expressed as ratios of full‐time positions per hospital bed, depending on the type of ward. In the Czech Republic and Slovakia, nurse staffing is primarily determined based on the minimum number of staff per shift and the number of patients assigned to one nurse. Differences in the organization of nursing care may influence both the scope of professional responsibilities and the level of workload among staff. Furthermore, variations in staffing models can affect workload levels, and European studies indicate that a higher number of patients per nurse are associated with increased hospital mortality and a higher risk of professional burnout [20].

The aim of this study was to assess the level of workload among nurses employed in Poland, Slovakia, and the Czech Republic, and to identify sociodemographic factors influencing perceived organizational oppression and a perfectionistic work style.

## 2. Materials and Methods

### 2.1. Research Design

A multicenter cross‐sectional study was conducted among nurses employed in hospitals in Poland, Slovakia, and the Czech Republic. The study was carried out between September 2024 and March 2025. Participation was voluntary and anonymous. The study design and reporting of results were prepared in accordance with the Strengthening the Reporting of Observational Studies in Epidemiology (STROBE) guidelines for cross‐sectional observational studies—details are presented in Appendix 1.

### 2.2. Research Tools

The research tools included a proprietary questionnaire covering sociodemographic data and a standardized instrument: the Working Excessively Questionnaire (WEQ—KONOP).

#### 2.2.1. Proprietary Questionnaire

This self‐designed questionnaire consisted of seven items aimed at collecting variables related to sociodemographic characteristics such as gender, age, and education level. Regarding job‐related characteristics, the questionnaire gathered information about the respondent’s job position, total years of professional experience, length of service at the current institution, and work schedule.

#### 2.2.2. WEQ (KONOP)

The KONOP (WEQ) is a tool designed to assess excessive work involvement. Its items cover potential causes, risk factors, and possible consequences of overwork. This allows for an assessment of whether a person’s work involvement is excessive and helps identify contributing reasons. The final version of the questionnaire was developed through research conducted between 2007 and 2013 [21]. The tool comprises 65 statements. Based on factor analysis and content review, the questionnaire includes the following four subscales:

1. Loss of control over work (UKP)—16 items; Cronbach’s alpha: 0.89
2. Perfectionist work style (PSP)—18 items; Cronbach’s alpha: 0.83
3. General work attitudes (OPP)—19 items; Cronbach’s alpha: 0.90
4. Perceived organizational oppressiveness (SOO)—12 items; Cronbach’s alpha: 0.71

The first subscale, loss of control over work, includes items indicating symptoms of losing control over work across various domains (resulting from a conscious or unconscious internal compulsion). These symptoms reflect an inadequate approach to work, especially evident in emotional, psychophysical, and social dimensions. Scores range from 18 to 80 points.

The second subscale, perfectionist work style, evaluates how professional tasks are performed, emphasizing a perfectionist approach. It aims to identify individuals whose work style and worldview contribute to work overload. Scores range from 16 to 90 points.

The third subscale, general work attitudes, measures the degree to which respondents identify with normative beliefs about work—particularly the belief that a person’s value is defined by their work. Scores range from 19 to 95 points.

The fourth subscale, perceived organizational oppressiveness, assesses attitudes toward the current employing institution. It distinguishes between organizationally imposed duties and the intrinsic content of work. It also reflects whether the organization encourages excessive workload and seeks to influence various aspects of employees’ lives. Scores range from 12 to 60 points.

The questionnaire is intended not only to determine the level of excessive workload but also to identify its potential negative consequences [22].

For the purposes of the study, a cultural adaptation of the questionnaire was carried out for respondents from Slovakia and the Czech Republic. The adaptation process included translation of the instrument into Slovak and Czech, as well as verification of the translation accuracy by experts in the field of nursing. The study was conducted with the permission of the author of the KONOP questionnaire.

The KONOP questionnaire (WEQ) is a tool developed and validated in Poland. For the purposes of the present study, the instrument was culturally adapted for respondents in Slovakia and the Czech Republic, including forward–backward translation and an assessment of content equivalence by experts in the field of nursing.

Due to the nature of the study, a full psychometric validation of the instrument was not conducted in each country, and the reported Cronbach’s alpha coefficients refer to the original validation carried out in Poland. The KONOP questionnaire was used in the present study with the author’s permission.

### 2.3. Participants

The study was conducted in public hospitals in Poland, the Czech Republic, and Slovakia. In each country, the study was proposed to be carried out among nurses employed in three different hospitals. The facilities were selected using purposive sampling, based on the consent of hospital management to participate in the study. In each hospital, questionnaires were distributed across wards representing various clinical profiles. This approach aimed to increase the diversity of the study sample and to account for different organizational conditions of nursing work in the countries examined.

The inclusion criteria were as follows:

• employment as a nurse in a hospital ward,
• active professional practice during the study period,
• provision of informed consent to participate in the study.

The exclusion criteria were as follows:

• employment in primary healthcare,
• exclusive work in outpatient care,
• lack of consent to participate in the study.

The questionnaires were distributed to cooperating hospitals with a request for voluntary and anonymous completion. The sample size (*n* = 300 in each country) was determined primarily on the basis of respondent availability, the organizational feasibility of the study, and the need to ensure comparability between countries. The adopted sample size is consistent with similar cross‐sectional studies conducted among nursing personnel.

A total of 774 respondents participated in the study, including 260 nurses from Poland, 256 from Slovakia, and 258 from the Czech Republic. The response rates were as follows:

• 86.6% in Poland,
• 85.3% in Slovakia,
• 86.0% in the Czech Republic.

All returned questionnaires were completed correctly and were included in the subsequent statistical analysis.

### 2.4. Statistical Analysis

The primary statistical test used in the analyses was the chi‐square test for independence. To measure the strength of associations, the Phi coefficient and Cramér’s *V* were used. The Mann–Whitney *U* test was applied for nonparametric assessment of differences. Correlations between ordinal or continuous variables were analyzed using Spearman’s rho coefficient. Statistical analysis was performed using IBM SPSS Statistics Version 26.0 with the exact tests module. All associations, correlations, and differences were considered statistically significant at *p* ≤ 0.05.

## 3. Results

### 3.1. Results of the Proprietary Questionnaire

In the Polish group, all respondents held higher education degrees. Among Slovak respondents, 24.6% had completed secondary education (medical high school), while the remaining participants held higher education degrees. In the Czech group, 140 respondents (54.3%) had secondary education, 100 (38.8%) held a bachelor’s degree in nursing, and 18 (7.0%) had completed higher education (master’s degree). A comparable number of nurses (217 and 218) in all groups worked as ward nurses in direct contact with patients. The majority of Polish and Slovak nurses worked 12‐h shifts (day and night). Detailed demographic data of the participants are presented in Table 1.

**TABLE 1:** Characteristics of the study group.

Variable		Polish nurses (*N* = 260)		Slovak nurses (*N* = 256)		Czech nurses (*N* = 258)	
		*N*	%	*N*	%	*N*	%
Gender	Female	238	91.5	236	92.2	248	96.1
	Male	22	8.5	20	7.8	10	3.9

Age (years)	Below 25	32	12.3	14	5.5	27	10.5
	25–35	68	26.2	47	18.4	57	22.1
	36–45	52	20.0	68	26.6	66	25.6
	46–55	98	37.7	77	30.1	77	29.8
	Above 55	10	3.8	50	19.5	31	29.8

Education	Secondary education	—	—	63	24.6	140	54.3
	Bachelor’s degree in nursing	202	77.7	101	39.5	100	38.8
	Master’s degree in nursing	58	22.3	92	35.9	18	7.0

Total work experience (years)	Below 5	96	36.9	39	15.2	43	16.7
	5–10	30	11.5	41	16.0	45	17.4
	11–20	42	16.2	58	22.7	59	22.9
	21–30	64	24.6	51	19.9	65	25.2
	Above 30	28	10.8	67	26.2	46	17.8

Tenure at current workplace (years)	Below 5	116	44.6	69	27.0	91	35.3
	5–10	54	20.8	72	28.1	68	26.4
	11–20	38	14.6	58	22.7	68	26.4
	21–30	36	13.8	34	13.3	26	10.1
	Above 30	16	6.2	23	9.0	5	1.9

Position	Ward nurse	218	83.8	217	84.8	222	87.2
	Coordinating nurse	22	8.5	24	9.4	26	10.1
	Head nurse	20	7.7	15	5.9	7	2.7

Work schedule	Single shift—7 h 35 min	66	25.4	72	28.1	63	24.4
	Shift work—8‐h shifts	8	3.1	21	8.2	2	0.8
	Shift work—12‐h shifts	172	66.2	128	50.0	125	48.4
	Mixed schedule	14	5.4	35	13.7	68	26.4

Compared to the Slovak group, Polish respondents more frequently held bachelor’s degree and less frequently held master’s degree or secondary‐level education. In contrast, secondary education was most common among Czech participants. The relationship was statistically significant (*p* ≤ 0.001) and demonstrated a strong association (Cramér’s *V* = 0.398).

There was no statistically significant relationship between gender and nationality group (*p* > 0.05). Due to the gender imbalance (overwhelmingly female respondents), this variable was excluded from further analysis. Older respondents were more prevalent in the Slovak group, while younger participants were more common in the Polish group. This relationship was statistically significant (*p* ≤ 0.001) and showed a moderate strength (Cramér’s *V* = 0.286) (see Table 2).

**TABLE 2:** Relationship between age and survey group.

Variables			Group			Total
			PL	SK	CZ	
2. Age (years)	Below 25	*N*	32	14	27	73
		%	12.3	5.5	10.5	9.4
	25–35	*N*	68	47	57	172
		%	26.2	18.4	22.1	22.2
	36–45	*N*	52	68	66	186
		%	20.0	26.6	25.6	24.0
	46–55	*N*	98	77	77	252
		%	37.7	30.1	29.8	32.6
	Above 55	*N*	10	50	31	91
		%	3.8	19.5	29.8	11.8

Total		*N*	260	256	258	774
		%	100.0	100.0	100.0	100.0

Cramer’s *V*	0.167	43.249	8	≤ 0.001	≤ 0.001	
Coefficient	value	Chi‐square	*df*	*p*	Monte Carlo *p*	

In comparison with the Slovak group, the Polish group had a higher percentage of respondents with a bachelor’s degree and a lower percentage with a master’s degree. The association was statistically significant (*p* ≤ 0.001) with strong strength (Cramér’s *V* = 0.450) (see Table 3).

**TABLE 3:** Relationship between education level and survey group.

Variable			Group			Total
			PL	SK	CZ	
3. Education	Secondary education	*N*	0	63	140	203
		%	0.0	24.6	54.3	26.2
	Bachelor’s degree in nursing	*N*	202	101	100	403
		%	77.7	39.5	38.8	52.1
	Master’s degree in nursing	*N*	58	92	18	168
		%	22.3	35.9	7.0	21.7

Total		*N*	260	256	258	774
		%	100.0	100.0	100.0	100.0

Cramer’s *V*	0.398	244.876	4	≤ 0.001	≤ 0.001	
Coefficient	value	Chi‐square	*df*	*p*	Monte Carlo *p*	

Using the Kruskal–Wallis test, only one statistically significant difference was found in the Czech group: perceived organizational oppressiveness varied significantly based on job position (*p* ≤ 0.01). Higher scores were reported by head nurses, followed by coordinating nurses and ward nurses.

A longer total work experience was more common among Slovak and Czech respondents compared to Polish participants. This relationship was statistically significant (*p* ≤ 0.001), with a slight strength (Cramér’s *V* = 0.195). Likewise, a longer tenure at the current workplace was more frequent among Slovak respondents (*p* ≤ 0.001, Cramér’s *V* = 0.153).

Polish respondents were more likely to work 12‐h shifts compared to Slovak and Czech respondents, but were less likely to work mixed shifts. This association was statistically significant (*p* ≤ 0.001), with a weak strength (Cramér’s *V* = 0.207).

The observed differences in age structure, level of education, and length of professional experience between the studied groups may partially influence the perception of workload in the respective countries.

### 3.2. Results of the Standardized Instrument—KONOP (WEQ)

In the surveyed group of Polish nurses, the mean score for loss of control over work was 42.96 (min. 16, max. 80), for perfectionistic work style 66.56 (min. 18, max. 90), for general attitudes toward work 47.65 (min. 19, max. 95), and for perceived organizational oppressiveness 35.18 (min. 12, max. 60).

In the surveyed group of Slovak nurses, the mean score for loss of control over work was 40.77 (min. 16, max. 80), for perfectionistic work style 63.28 (min. 18, max. 90), for general attitudes toward work 48.95 (min. 19, max. 95), and for perceived organizational oppressiveness 33.21 (min. 12, max. 60).

Comparable results to those of Slovak nurses were obtained in the group of Czech nurses. The mean score for loss of control over work was 40.73 (min. 21, max. 67), for perfectionistic work style 60.69 (min. 40, max. 85), for general attitudes toward work 46.02 (min. 27, max. 70), and for perceived organizational oppressiveness 30.35 (min. 13, max. 44).

Analysis using the Mann–Whitney *U* test showed that the results in three KONOP domains differed significantly depending on the study group (*p* ≤ 0.001). Nursing staff from Poland, compared to those from Slovakia and the Czech Republic, demonstrated slightly higher scores in loss of control over work, perfectionistic work style, and perceived organizational oppressiveness. In contrast, higher scores for general attitudes toward work were observed among respondents from Slovakia, followed by those from the Czech Republic, and the lowest among respondents from Poland (Table 4).

**TABLE 4:** KONOP questionnaire results.

Group		Loss of control over work (16–80)	Perfectionist work style (18–90)	General work attitudes (19–95)	Perceived organizational oppressiveness (12–60)
PL	Mean	42,96	66.56	47.65	35.18
	Median	42.00	68.50	47.50	35.00
	Mean rank	426.85	479.73	389.48	468.90
	Frequency (*N*)	260	260	260	260
	Standard deviation	10.52	13.18	11.13	6.70
	Minimum	19.00	18.00	20.00	16.00
	Maximum	69.00	88.00	85.00	53.00

SK	Mean	40.77	63.28	48.95	33.21
	Median	40.00	63.50	48.00	33.00
	Mean rank	366.13	376.30	425.37	401.26
	Frequency (*N*)	256	256	256	256
	Standard deviation	7.85	8.81	9.14	4.51
	Minimum	18.00	28.00	20.00	17.00
	Maximum	69.00	87.00	87.00	47.00

CZ	Mean	40.73	60.69	46.02	30.65
	Median	40.00	61.00	45.00	31.00
	Mean rank	369.05	305.67	347.92	291.81
	Frequency (*N*)	258	258	258	258
	Standard deviation	9.66	7.76	7.47	4.66
	Minimum	21.00	40.00	27.00	13.00
	Maximum	67.00	85.00	70.00	44.00

Total	Mean	41.49	63.52	47.54	33.02
	Median	41.00	64.00	47.00	33.00
	Mean rank	774	774	774	774
	Frequency (*N*)	9.46	10.47	9.43	5.69
	Standard deviation	18.00	18.00	20.00	13.00
	Minimum	69.00	88.00	87.00	53.00

Kruskal–Wallis *H*		Maximum	79.540	15.476	83.005
*p*		0.002	≤ 0.001	≤ 0.001	≤ 0.001
*p* (Monte Carlo)		0.002	≤ 0.001	≤ 0.001	≤ 0.001

Abbreviations: CZ, Czech nurses; PL, Polish nurses; SK, Slovak nurses.

The observed differences between the studied countries suggest a possible influence of organizational and systemic factors related to the functioning of healthcare systems in each country. A detailed interpretation of these relationships is presented in the Discussion section.

In the Polish group, longer tenure at the current workplace correlated significantly with higher scores on the *loss of control over work* subscale (*p* < 0.05), although the strength of correlation was weak (Spearman’s rho = 0.131). Age, education level, and total work experience were not significantly correlated with any KONOP subscales (*p* > 0.05) (see Table 5).

**TABLE 5:** Correlation between sociodemographic variables and KONOP subscales—Polish group (*N* = 260).

Polish nurses			Age	Education	Total work experience	Tenure at current workplace
Spearman’s rho	Loss of control over work (16–80)	Correlation coefficient	−0.074	0.024	0.011	0.131
	Perfectionist work style (18–90)	Correlation coefficient	−0.045	0.033	0.074	0.055
	General work attitude (19–95)	Correlation coefficient	0.015	−0.095	0.086	0.091
	Perceived organizational oppressiveness (12–60)	Correlation coefficient	−0.042	0.028	0.030	0.075

Among Slovak nurses, higher education levels correlated with increased scores in *loss of control over work* and *perfectionist work style*, as did total work experience (*p* < 0.05). However, the correlations were weak (low Spearman’s rho values). Age and tenure at the current workplace showed no statistically significant correlation with KONOP scores (see Table 6).

**TABLE 6:** Correlation between sociodemographic variables and KONOP subscales—Slovak group (*N* = 256).

Slovak nurses			Age	Education	Total work experience	Tenure at current workplace
Spearman’s rho	Loss of control over work (16–80)	Correlation coefficient	0.071	0.148	0.051	0.079
	Perfectionist work style (18–90)	Correlation coefficient	0.081	0.124	0.140	0.102
	General work attitude (19–95)	Correlation coefficient	0.088	0.023	0.083	0.046
	Perceived organizational oppressiveness (12–60)	Correlation coefficient	0.078	−0.065	0.073	0.111

In the Czech group, no statistically significant correlations were found between age, education level, total work experience, or tenure at the current workplace and any KONOP subscales (*p* > 0.05) (see Table 7).

**TABLE 7:** Correlation between sociodemographic variables and KONOP subscales—Czech group (*N* = 258).

Czech nurses			Age	Education	Total work experience	Tenure at current workplace
Spearman’s rho	Loss of control over work (16–80)	Correlation coefficient	−0.076	0.081	−0.098	−0.039
	Perfectionist work style (18–90)	Correlation coefficient	−0.121	0.084	−0.103	−0.102
	General work attitude (19–95)	Correlation coefficient	0.027	−0.009	−0.052	0.003
	Perceived organizational oppressiveness (12–60)	Correlation coefficient	−0.023	0.023	−0.115	−0.072

The observed differences between the studied countries are discussed in detail in the Discussion section.

## 4. Discussion

The aim of this study was to assess the level of excessive workload among nurses employed in Poland, Slovakia, and the Czech Republic, as well as to identify sociodemographic factors associated with its perception. The obtained results indicate that the level of perceived workload differs between the studied countries, which may be related to organizational conditions within healthcare systems, including staff shortages, the organization of shift work, and the availability of resources supporting nursing staff.

In light of the JD‐R model, a high level of job demands combined with limited organizational resources may lead to increased occupational stress and a sense of excessive workload among nurses. The findings confirm that both individual factors and organizational conditions may influence how nursing staff perceive workload.

The results of this study align with a growing body of international research indicating that excessive workload among nurses constitutes a significant challenge for healthcare systems and may have consequences for both staff well‐being and the quality and safety of patient care. The observed differences between the studied countries may result from varying systemic conditions related to the organization of nursing work and workforce policies within healthcare systems. In many Central European countries, a shortage of nursing staff is observed, leading to an increased number of patients per nurse and greater time pressure in clinical work. European studies indicate that increased workload and insufficient nurse staffing are associated with higher levels of professional burnout and a deterioration in the quality of patient care [20, 23]. Differences in workload levels may also stem from variations in nursing education models, employment structures, and the organization of shifts across countries.

Similar findings have been reported by other authors, highlighting the relationship between excessive workload and professional burnout, decreased job satisfaction, and an increased risk of medical errors [15]. Kowalczuk et al. analyzed the level of excessive workload, and their results confirmed the presence of this phenomenon among the studied group of nurses. The highest burden was associated with a perfectionistic work style, while the lowest burden was related to general attitudes toward work [24].

The consistency of the obtained results with international reports indicates that the problem of excessive workload among nurses is universal in nature and is not limited solely to the healthcare systems of Central European countries.

In another study, 85% of nurses reported being overworked, with 30% indicating a moderate to severe degree of overwork. Sociodemographic characteristics and the work environment do not always demonstrate a clear influence on the level of nurses’ workload [25]. This is partially confirmed by the findings of the present study, although significant differences between the studied countries were observed in the analyzed sample.

The obtained results are consistent with studies conducted in Poland, which indicate a significant relationship between occupational stress, work overload, and job satisfaction among nursing staff. In the studies by Kwiecień‐Jaguś et al., higher levels of stress were shown to be associated with lower job satisfaction, particularly in the context of interpersonal conflicts and inadequate remuneration [26, 27]. Similar observations were reported by Stępień and Szmigiel, who indicated that the main sources of stress in nurses’ work include excessive duties, time pressure, poor work organization, and constant supervision, all of which increase the risk of medical errors and reduce work motivation [28]. In turn, Kwak et al., in a meta‐analysis of studies, emphasized that the problem of occupational stress and coping strategies among nurses remains insufficiently explored, which justifies the need for further research using standardized instruments [29]. Guz et al., on the other hand, demonstrated that inadequate staffing standards and personnel shortages contribute to an increased psychophysical burden on nurses and may reduce patient care safety [30]. These findings strengthen the interpretation of the obtained results and underscore the need for organizational measures aimed at improving the working conditions of nursing staff.

Available research on nursing workload frequently focuses on its impact on care quality and professional burnout. Diehl et al. emphasized that workload in the nursing profession significantly affects nurses’ health, thereby justifying the need to explore the underlying contributing factors [31]. Similarly, Maghsoud et al. concluded that excessive workload negatively influences the quality of nursing care [5].

Miller and Hemberg indicate that the analysis of workload should primarily be conducted from the perspective of nurses providing direct patient care, as it is at this level that the greatest organizational and psychosocial consequences of excessive workload become evident [32]. This is supported by the study of Havaei and MacPhee, who found higher workload levels at the frontline nursing position [33].

The results of the present study also point to the significant role of sociodemographic factors in the perception of workload. Among Polish nurses, an increase in length of service at the current workplace was associated with higher scores in loss of control over work. This may suggest that prolonged functioning in a demanding work environment can lead to the accumulation of occupational stress and an increased sense of overload. At the same time, some studies indicate that professional experience may serve as a protective factor, enhancing coping skills in relation to stress and job demands [34].

An important aspect of interpreting the results is also the impact of excessive workload on the quality of patient care. The literature emphasizes that an increased number of patients per nurse may lead to reduced time spent with each patient, a higher number of missed nursing care interventions, and an increased risk of adverse events in healthcare [35]. Studies by Aiken et al. demonstrated that a higher patient‐to‐nurse ratio is significantly associated with increased hospital mortality and poorer patient outcomes [20]. In the context of the present study, this highlights the importance of monitoring workload levels as a component of ensuring patient safety.

The obtained results can also be interpreted in light of psychosocial work models, such as the JD‐R model. According to this approach, high job demands—such as time pressure, responsibility for patients’ lives, and a large number of tasks—combined with insufficient organizational resources may lead to work overload and emotional exhaustion [16]. In the nursing environment, these factors may be further intensified by the nature of work requiring high emotional involvement and rapid clinical decision‐making.

An important element of the findings is also the relationship between a perfectionistic work style and perceived workload. A perfectionistic approach to professional duties may increase work engagement; however, in the long term, it may also contribute to overload and increase the risk of professional burnout. Similar relationships have been observed in other studies on nursing staff, which demonstrated an association between high job demands, professional perfectionism, and increased stress and occupational fatigue [31].

In the present study, higher scores in general attitudes toward work were observed among nurses from Slovakia, while lower scores were noted among respondents from the Czech Republic and Poland. At the same time, nurses from Poland achieved higher scores in loss of control over work, perfectionistic work style, and perceived organizational oppressiveness.

Ditlopo et al. found that nurses evaluated their workload negatively, linking it to job dissatisfaction and an increased desire to leave the profession. They also noted its adverse effect on job performance and care quality [34]. Kang and Bang demonstrated that organizational oppression, hostile work environments, and patient aggression increase stress and workload, negatively affecting nurses’ physical and mental health. High workload frequently leads to burnout, lower job satisfaction, and reduced care quality [36], findings that are echoed in studies by other authors [37].

In the present study, among Polish nurses, an increase in length of service at the current workplace was associated with higher levels of loss of control over work. Negative correlations between age and work experience with overload suggest a buffering role of experience. Psychosocial factors [4] are associated with perceived organizational oppressiveness—manifested as a sense of limited autonomy, excessive control, and lack of recognition. Strong positive relationships between loss of control over work (UKP), perfectionistic work style (PSP), and organizational oppressiveness (OO) indicate their mutual reinforcement and the risk of a vicious cycle of perfectionism and loss of control.

The findings of Alzoubi et al. demonstrated that perceived workload among nurses increases with age [38], including in the psychological domain [39]. Yuan et al. confirmed that nurses work under a high psychological burden (with 54% experiencing such strain), highlighting the urgent need for interventions aimed at reducing this burden [40]. Surendran et al. showed that nursing work requiring high cognitive and mental load may reduce patient safety and lead to professional burnout [41]. The study by Nemati‐Vakilabad et al. indicated that increased nursing workload was significantly associated with higher levels of both acute and chronic fatigue. Moreover, effective teamwork among nurses—including trust, team orientation, support, shared mental models, and team leadership—was associated with reduced fatigue levels [42], which also represents an important implication of the present study.

From a nursing management perspective, the findings indicate the need to implement organizational measures aimed at reducing excessive workload among staff. Nursing managers play a key role in monitoring workload levels, planning nurse staffing, and creating a work environment conducive to employee well‐being [43]. The implementation of workload monitoring systems, the development of mentoring programs for less experienced nurses, and the promotion of teamwork may contribute to improving working conditions and enhancing the quality of patient care.

In summary, the obtained results indicate that excessive workload constitutes a significant challenge for nurses working within healthcare systems in Central European countries. The observed differences between the studied countries may reflect variations in organizational conditions, including the availability of human resources, the organization of work, and institutional support for nursing staff. In the context of the JD‐R model, these findings highlight the importance of balancing job demands with adequate organizational resources, which can protect employees from the negative consequences of excessive workload. From the perspective of healthcare workforce management, the role of nursing leaders and managers is particularly important in monitoring workload levels and implementing organizational measures aimed at improving working conditions and professional well‐being. Such actions may contribute not only to increased job satisfaction among staff but also to improved quality and safety of patient care.

The obtained results indicate a persistent lack of significant systemic changes in the working conditions of nurses over many years, particularly in postsocialist countries. This underscores the need to implement organizational and systemic measures aimed at improving working conditions, reducing workload, and increasing the professional satisfaction of nursing staff.

## 5. Conclusion

The conducted study demonstrated that the level of perceived workload among nurses varies depending on the country as well as selected sociodemographic factors, such as age, education, and length of professional experience. The highest levels of excessive workload were observed among nurses from Poland, which may indicate specific organizational challenges within the healthcare system in this country. The most burdensome dimensions of work were identified as a perfectionistic work style and perceived organizational oppressiveness. The obtained results highlight the importance of organizational measures aimed at reducing excessive workload among nurses. From the perspective of healthcare workforce management, particular importance should be placed on monitoring workload levels, appropriate nurse staffing, and creating a work environment that supports the professional well‐being of staff. The implementation of such measures may contribute to improved job satisfaction among nurses, a reduction in the risk of professional burnout, and enhanced quality and safety of patient care.

## 6. Limitations of the Study

The results of the conducted study have several limitations. The study included nurses from Poland, the Czech Republic, and Slovakia, and the sample size may limit the generalizability of the findings. The study has a cross‐sectional design, which precludes causal inference, while the purposive sampling method and the use of self‐report instruments may limit the representativeness of the sample and involve a risk of subjective assessment bias. The study included a sample from three Central European countries, which may limit the generalizability of the findings to other healthcare systems. An additional limitation is that the KONOP questionnaire has been fully psychometrically validated only in Poland, whereas for the Czech Republic and Slovakia, only cultural adaptation was performed, without full psychometric validation, which may affect the comparability of results between countries. Furthermore, the study focused solely on the impact of sociodemographic factors on workload. Further multicenter studies are necessary to enable broader generalization and to deepen the understanding of this issue.

## 7. Implications for Nursing Management

Consideration should be given to implementing mentoring and adaptation programs to strengthen the team’s organizational and emotional competencies, as well as to

• systematically monitor workload (e.g., using KONOP);
• plan schedules flexibly and balance workload between teams;
• introduce psychoeducational activities addressing perfectionism and self‐regulation;
• build an organizational culture based on partnership, recognition, and trust;
• define clear career paths and competence‐based rewards to enhance motivation and reduce work overload;
• regularly monitor the level of identification with the professional role, as well as absenteeism and turnover indicators, to enable ongoing adjustment of interventions and optimization of nurses’ working conditions.

## Author Contributions

Katarzyna Tomaszewska, Bożena Majchrowicz, Helena Kadučáková, Anna Krátká, Erika Durejova, and Krystyna Kowalczuk were the principal investigators for the study, generated the idea, and designed the study. Katarzyna Tomaszewska, Bożena Majchrowicz, and Krystyna Kowalczuk were the primary writers of the manuscript and approved all changes. Katarzyna Tomaszewska, Bożena Majchrowicz, and Krzysztof Kalita supported the data input and data analysis. Katarzyna Tomaszewska, Bożena Majchrowicz, Helena Kadučáková, Anna Krátká, Erika Durejova, and Krystyna Kowalczuk supported the data collection. All authors were involved in literature review, developing, editing, reviewing, and providing feedback for this manuscript.

## Funding

This research received no external funding.

## Disclosure

All authors approved the final version to be published.

## Ethics Statement

The study was conducted in accordance with the Declaration of Helsinki and received ethical approval from the Bioethics Committee at the State Academy of Applied Sciences in Przemyśl, Poland (KBPANS/3/2024); the Bioethics Committee of the Central Military Hospital SNP in Ružomberok, Slovakia (UNV‐40‐29/224); and the Bioethics Committee of Tomáš Baťa Regional Hospital in Zlín, Czech Republic (approval dated December 18, 2024).

## Consent

Consent for publication was obtained from all study participants.

## Conflicts of Interest

The authors declare no conflicts of interest.

## Supporting Information

Additional supporting information can be found online in the Supporting Information section.

## Supporting information


**Supporting Information** STROBE Checklist: a checklist of items required for reporting observational cross‐sectional studies in accordance with the STROBE guidelines (Strengthening the Reporting of Observational Studies in Epidemiology), applied in the present study.

## Data Availability

The data that support the findings of this study are available from the corresponding author upon reasonable request.
