# Supplementary material for: Workload Pressure Among Nurses in Central Europe: Implications for Human Resource Management in Poland, the Czech Republic, and Slovakia
Source: J Nurs Manag. 2026 May 27;2026:9303525. doi: 10.1155/jonm/9303525 (PMC13215978; doi:10.1155/jonm/9303525)
Supplement: Supplementary file 1 — Supporting Information STROBE Checklist: a checklist of items required for reporting observational cross‐sectional studies in accordance with the STROBE guidelines (Strengthening the Reporting of Observational Studies in Epidemiology), applied in the present study. [file JONM-2026-9303525-s001.docx]

**Table - STROBE Checklist**

| **Section** | **STROBE item** | **Description** | **Location in the manuscript** |
| --- | --- | --- | --- |
| **Title and abstract** | 1 | Indication of the study type in the title or abstract | Title / Abstract |
|  | 2 | Concise summary of methods and results | Abstract |
| **Introduction** | 3 | Scientific rationale and background of the study | Introduction |
|  | 4 | Clearly stated study objective | Introduction (last paragraph) |
| **Methods** | 5 | Description of the study design | Methods - Study design |
|  | 6 | Study setting, time, and conditions | Methods - Study setting |
|  | 7 | Inclusion and exclusion criteria | Methods - Participants |
|  | 8 | Data sources and methods of measurement | Methods - Research instruments |
|  | 9 | Potential sources of bias | Methods - Limitations |
|  | 10 | Justification of sample size | Methods - Sample selection |
|  | 11 | Description of variables | Methods - Variables |
|  | 12 | Statistical methods | Methods - Statistical analysis |
| **Results** | 13 | Number of participants at each stage | Participants/Results |
|  | 14 | Characteristics of the study group | Results - Descriptive data |
|  | 15 | Data on variables | Results |
|  | 16 | Main results | Results |
|  | 17 | Additional analyses | Results (if applicable) |
| **Discussion** | 18 | Summary of key findings | Discussion |
|  | 19 | Study limitations | Discussion - Limitations |
|  | 20 | Interpretation of results | Discussion |
|  | 21 | Generalizability of the results | Discussion |
| **Other information** | 22 | Funding sources | Funding section |
|  | 23 | Bioethics committee approval | Methods / Statements |
